# 3D-Printed Complete Dentures: A Review of Clinical and Patient-Based Outcomes

**DOI:** 10.7759/cureus.69698

**Published:** 2024-09-19

**Authors:** Mohamed H Abdelnabi, Amal A Swelem

**Affiliations:** 1 Oral and Maxillofacial Prosthodontic Department, King Abdulaziz University, Jeddah, SAU; 2 Prosthodontic Department, Cairo University, Cairo, EGY

**Keywords:** ohrqol, patient satisfaction, clinical outcomes, digital dentures, 3d printed dentures

## Abstract

There has recently been an increasing trend to shift the fabrication of complete dentures from conventional to digital workflows to shorten the treatment time and increase patient comfort and satisfaction. Digital fabrication of complete dentures can be achieved either by a subtractive process (milling) or by an additive technique (3D printing). The milling process offers numerous advantages; however, they require large-size production machines and are associated with low production efficiency, increased cost, limited block size, and a considerable waste of material. On the other hand, 3D printing technology can potentially offer the benefits of lower manufacturing and equipment costs, good surface details, and lower material waste. Hence, 3D printing is being considered lately by some researchers as a valid choice for manufacturing digital dentures. Therefore, the aim of the current review was to identify and highlight studies on 3D-printed dentures, mainly those investigating clinical and patient-centered outcomes. A search was conducted using the databases PubMed/MEDLINE, Cochrane Library, Embase, and Google Scholar. After applying the inclusion and exclusion criteria, a total of 16 studies that investigated clinical outcomes (masticatory efficiency, biting force, retention and stability, computerized occlusal analysis, and post-insertion maintenance) as well as patient-based outcomes (patient satisfaction, oral health-related quality of life (OHRQoL), patient-related complications, patient preference, and willingness to pay) were included. After a thorough review and discussion of these articles, it could be concluded that 3D printing of complete dentures offers many advantages from both a clinical and patient-based perspective. Retention and comfort with 3D-printed dentures were found to be comparable or even superior to conventional dentures. Moreover, retention of 3D-printed dentures constructed from conventional impressions and digitized casts demonstrated improved retention when compared to a protocol adopting intraoral scanning (digital impressions). Masticatory efficiency, biting force, OHRQoL, and patient satisfaction with 3D-printed dentures varied and were inconsistent among the included studies. Most of the studies reported positive results in the different domains and assessed aspects, while others reported some concerns (especially in terms of aesthetics and phonetics). With regard to post-insertion maintenance, printed dentures showed comparable results to conventional dentures in the short term. The technique seems promising with numerous benefits; however, further clinical research with larger sample sizes and longer follow-up periods is still needed to confirm these conclusions and address the potential concerns.

## Introduction and background

Complete dentures have been and are still considered the most commonly used treatment option for completely edentulous patients [1]. However, their conventional fabrication is usually associated with a complex sequence of clinical and laboratory steps that are cost-intensive and time-consuming [2,3]. Other drawbacks of conventionally constructed dentures are related to the characteristics of polymethylmethacrylate (PMMA) resin, such as polymerization shrinkage, low mechanical strength, and porosity [4,5]. More importantly, they require frequent patient visits and hence longer chair time [5]. As a result, there has been an increasing demand to streamline the workflow and shift to digitally fabricated dentures to shorten the treatment time and increase patient comfort and satisfaction [3,6,7]. Digitally constructed dentures can reduce the number of visits required to complete the process (up to two appointments in some cases), reduce the workload for technicians, and have been found to have comparable or better retention and base fit than conventional heat-polymerized dentures [8,9]. Furthermore, they have shown favorable short-term clinical performance, positive patient-related outcomes, and reasonable cost and time effectiveness [10-12].

According to the Glossary of Prosthodontic Terms, digitally fabricated dentures are complete dentures created using computer-aided design and computer-aided manufacturing (CAD-CAM) technology [13]. They can be achieved either by a subtractive process (milling) or an additive technique (3D printing) [11]. The subtractive process involves milling dentures out of pre-polymerized, commercially produced PMMA blanks that have been manufactured under controlled conditions and high pressure. Additive techniques comprise the deposition of liquid resins on a support structure (layer-by-layer), followed by curing with ultraviolet light, visible light, heat, or laser [5,14,15]. It has been reported that milled dentures demonstrate superior mechanical and surface properties, good color stability and biocompatibility, as well as a high degree of polymerization and fracture resistance [16-20]. However, the milling process has several disadvantages including the large size of production machines, high time consumption, low production efficiency, increased cost, limited block size, and considerable waste of material (an entire blank is used to mill a denture base). The generated waste (cutting debris) also raises environmental concerns [5,7]. Accordingly, many researchers are now considering additive manufacturing methods as a preferred alternative [5]. 3D printing technology can potentially offer advantages such as lower manufacturing and equipment costs, speed, good surface details, and lower material waste [7,11,21]. Despite some concerns about the aesthetics of printed dentures, it is believed that rapid technological developments in 3D printers and newer resins may overcome this shortcoming in the near future. Hence, 3D printing might possibly be a valid choice for manufacturing digital dentures [11], which explains why more studies have been conducted on 3D-printed dentures recently. From this perspective, the aim of the current review was to identify and highlight the studies focussing mainly on clinical and patient-centered outcomes.

## Review

A search was conducted for articles related to 3D-printed complete dentures. The searched databases were PubMed/MEDLINE, Cochrane Library, Embase, and Google Scholar. Keywords were used (digital, CAD/CAM, 3D-printed, printed, digitally manufactured, rapid prototyping, digitally fabricated) with the Boolean operator AND (removable complete dental prostheses, dentures, complete dentures, removable prostheses). Systematic reviews related to the topic in hand were also critically revised and referenced studies in these reviews were considered. A manual search was conducted when necessary.

Inclusion criteria involved articles available in full text and published in English and clinical trials that investigated clinical outcomes (masticatory efficiency, biting force, retention and stability, computerized occlusal analysis, and post-insertion maintenance) as well as patient-based outcomes (patient satisfaction, oral health-related quality of life, patient-related complications, patient preference, and willingness to pay). Studies not yet published or not available in full text and articles published in languages other than English as well as in-vitro studies or those that did not align with the aim of the current review were excluded.

Abstracts of the articles that resulted from the initial search were screened and filtered for full text reading. After applying the inclusion and exclusion criteria, a total of 16 studies were included in this review. Details of the included articles [5,7,11,21-33] are presented in Table 1.

**Table 1:** Details of the included articles PMMA: Polymethylmethacrylate; nanoTiO_2_: Nanosized titanium dioxide; PDs: 3D-printed dentures; CDs: Conventional dentures; OHIP-EDENT: Oral health impact profile for edentulous patients; OHIP-EDENT-J: Oral health impact profile for edentulous patients - Japanese version; OHRQoL: Oral health-related quality of life; CAD-CAM: Computer-aided design and computer-aided manufacturing

	Article	Study type	Sample size	Aim	Clinical outcomes	Patient-based outcomes	Follow-up period
1	Cristache et al. 2020 [5]	Clinical trial	35 participants	Assessed 3D-printed complete dentures manufactured using improved PMMA-​​​​​nanoTiO_2_	-	Patient satisfaction, OHIP-EDENT	Before denture insertion, at one week, 12 months, and 18 months
2	Srinivasan et al. 2021 [11]	Double-blind, randomized, crossover, clinical trial	15 participants	Compared newly constructed milled and 3D-printed complete removable dental prostheses to already existing old CDs	Denture quality (clinician’s evaluation), masticatory efficiency, maximum voluntary bite force, prosthodontic maintenance needs	Patient satisfaction, OHRQoL, willingness to pay analysis, patient preference (final choice)	Baseline (with old CDs), at one and six weeks after insertion of new digital dentures
3	Chaturvedi et al. 2021 [22]	Clinical trial	Five participants	Compared milled and PDs to conventional complete dentures. Each group was further divided into three sub-groups based on occlusion schemes (bilateral balanced, lingualized, and mono-plane)	Computerized occlusal force analysis	-	Assessment done at the time of denture insertion
4	Kim et al. 2021 [23]	Retrospective study	420 CDs (270 in axilla; 150 in mandible) and 216 PDs (130 in maxilla; 86 in mandible)	Compared PDs to CDs	Post-insertion maintenance (number of remakes, number of post-insertion adjustments, type and number of repairs)	Patient-reported complications	One year for both dentures (1-7-2015 to 30-6-2016 for CDs and 1-7-2017 to 30-6-2018 for PDs)
5	Ragheb and Ibrahim 2021 [24]	Clinical crossover study	27 participants	Compared milled and PDs to conventional complete dentures	Maximum biting force, masticatory efficiency	-	One-week, one-month, and three-month follow-ups with a two-week washout period between each denture type
6	Stilwell et al. 2021 [25]	Randomized, blinded survey	40 participants: undergraduate students (n=10), postgraduate residents (n=10), dental technicians (n=10), and elderly complete denture wearers (n=10)	Evaluated maxillary dentures fabricated by six different techniques (three conventional: flask-pack-press, injection molded and intrinsically colored natural gingiva finish before injection-molded, and three CAD-CAM methods: milled base with bonded prefabricated teeth, fully milled dentures including milled teeth, and fully PDs including printed teeth)	-	Appearance/aesthetics (extra-oral assessment)	-
7	Zohny et al. 2021 [26]	Clinical crossover study	Eight participants	Compared between PDs and CDs (maxillary only)	Denture retention	Patient satisfaction	At insertion and after one month for each denture type with a two-week washout period
8	Emera et al. 2022 [27]	Clinical crossover study	10 participants	Compared between PDs and CDs	Denture retention	-	Zero, three, and six-month follow-ups with a two-week washout period between the two denture types
9	Heikal et al. 2022 [28]	Randomized controlled clinical trial	48 participants	Compared milled and PDs to conventional complete dentures	Denture retention	Patient satisfaction	Zero, three, and six-month follow-ups
10	Ohara et al. 2022 [7]	Clinical crossover trial	20 participants but only 15 completed the study	Compared between PDs and CDs	Number of treatment visits, time required for denture fabrication, number of adjustment visits, time required for denture stabilization	Patient satisfaction and patient preference, OHIP-EDENT-J	Two and half years (from November 2017 to May 2020)
11	Al-Kaff and Al-Hamad 2023 [29]	Controlled clinical trial	20 participants	Compared PDs with intraoral scanning and hybrid cast digitization (laboratory scanning of the definitive casts) to conventional complete dentures	Clinician’s assessed denture quality	OHIP-EDENT	3-4 weeks after wearing each denture with only one day washout period
12	Faty et al. 2023 [30]	Clinical controlled crossover study	24 participants	Compared milled and printed maxillary denture bases to conventional ones	Retention of the denture base	-	Retention was evaluated after immersion in water for 24 hours
13	Osnes et al. 2023 [21]	Multi-center clinical trial	16 participants but only 14 completed the study	Compared PDs to CDs	Clinician-assessed retention, clinician-assessed stability	Patient assessment with regard to comfort, denture’s stability, and appearance, patient’s preference	Not specified
14	Kang et al. 2024 [31]	Randomized, single-blinded, cross-over clinical trial	Nine participants but only eight completed the study	Compared between PDs and CDs	Masticatory force, masticatory efficiency	Patient satisfaction	One month after wearing each denture type (washout period unspecified)
15	Maniewicz et al. 2024 [32]	Clinical controlled crossover study (Part I)	20 participants but only 19 completed the study	Compared milled and 3D-printed maxillary denture bases to CD bases	Retention of the denture base	Patient subjective evaluation of the denture bases	Before storage in the artificial saliva and after storage in saliva for 14 days
16	Chebib et al. 2024 [33]	Clinical controlled crossover study (Part II)	20 participants but only 19 completed the study	Compared milled and 3D-printed maxillary denture bases constructed from intraoral scans versus conventional impressions and cast digitization	Retention of the denture base	-	Before storage in the artificial saliva and after storage in saliva for 14 days

For a more organized, coherent, and comprehensible discussion, the review is divided into two main parts. Clinical outcomes are discussed in part 1 and patient-based outcomes are discussed in part 2.

1. Clinical outcomes

Biting Force and Masticatory Efficiency

Three of the included articles investigated the biting force and masticatory (chewing) efficiency of 3D-printed dentures as compared to conventional dentures [31] or to both conventional and milled dentures [11,24]. They were all cross-over studies, hence it could be assumed that the effect of patient-related variables (such as age, gender, and other factors) on the obtained results was eliminated to a great extent.

All three studies evaluated the masticatory efficiency using two color mixing tests. Nevertheless, the results reported by the three studies were inconsistent. It is worth mentioning, however, that Kang et al. [31], in contrast to the other two studies, used intraoral scanners (digital impressions) instead of scanning the master casts from border-molded conventional impressions. Moreover, Srinivasan et al. [11] used prefabricated commercial artificial teeth, while the other two studies [24,31] used 3D-printed teeth. Srinivasan et al. [11] reported insignificant differences in masticatory efficiency between the milled and 3D-printed dentures and old conventional dentures. They also added that it improved over time with both digitally fabricated dentures. Conflicting results were reported by the other two studies. Kang et al. [31] reported that masticatory efficiency with the conventional dentures was significantly superior to the 3D-printed dentures, while Ragheb and Ibrahim [24] reported the exact opposite and that digitally fabricated dentures (both milled and 3D-printed) demonstrated better masticatory efficiency than conventional dentures. Kang et al. [31] attributed their results to the possible challenges in achieving bilateral balanced occlusion in digital dentures. In addition, clinical remounting was not carried out. On the contrary, Ragheb and Ibrahim [24] justified the significant superiority of masticatory efficiency for the digitally fabricated dentures to the better adaptation achieved with CAD/CAM denture bases as compared to conventionally fabricated ones [34]. Consequently, better adaptation leads to improved retention, stability, and masticatory efficiency. Artiﬁcial teeth on a more retentive and stable base make the patient more comfortable and capable of exerting higher biting forces, thus enhancing masticatory efficiency [35].

As for the biting force, the results of two of the three studies [11,31] were consistent and reported insignificant differences in maximum biting force between digitally fabricated dentures and conventional dentures. The third study [24], however, reported that the maximum biting force was higher with digitally fabricated dentures than with conventionally fabricated ones. They attributed this to better adaptation, hence better stability, and retention achieved by the digital dentures. They supported their justification with the direct relationship that was reported in earlier studies between the level of maximum bite force and denture stability and retention [36,37]. Biting force values recorded for the 3D-printed dentures in the three studies varied. This could be attributed to the different measuring devices and procedures that were implemented. Srinivasan et al. [11] used a digital force gauge, Ragheb and Ibrahim [24] used the flexi-force sensor, while Kang et al. [31] used an occlusal force measuring system (Prescale).

It is worth mentioning that the sample size for two of the three studies [11,31] was relatively small. Could the results have been different with a larger sample size is a question that needs to be answered by further research.

Retention and Stability

Six of the included studies evaluated retention objectively [26-28,30,32,33]; three of them involved complete dentures [26-28], while denture bases only were considered in the other three [30,32,33]. Another three studies [11,21,29] used clinician-based assessments to evaluate retention and stability along with other factors.

Zohny et al. [26] compared the retention of maxillary printed to conventional dentures using a universal testing machine. Their results revealed that the printed dentures were significantly more retentive than the conventional dentures. Emera et al. [27] used a force meter to assess the retention of both maxillary and mandibular dentures. They reported insignificant differences between conventionally fabricated and 3D-printed dentures at all follow-up periods (zero, three, and six months). They also reported an increase in the mean retention force values by time for both denture types; however, this increase was statistically insignificant. Similar findings were presented in another study [28] that reported insignificant differences in retention values recorded for digitally fabricated dentures (milled and printed) as compared to conventional dentures. Retention values also increased over time for all three groups, but in contrast to the previous study, this increase was statistically significant by the end of the study period (six months).

Three studies [30,32,33] evaluated the retention of 3D-printed maxillary denture bases only. Faty et al. [30] reported that milled denture bases demonstrated significantly higher retention than printed and conventional denture bases with insignificant differences between the last two. The authors interpreted the superiority of the milled variety by the minimal dimensional changes as pre-polymerized blocks are used in the subtractive manufacturing process. On the other hand, conventional and even 3D-printed denture bases are liable to polymerization shrinkage with dimensional changes at varying degrees. The demounting of the dentures from the printing platform may also result in some deformation. These factors might partially compromise the adaptation of the denture bases to the underlying supporting tissues, justifying thereby the decreased retention in comparison to the milled bases. In partial disagreement with the previous study, Maniewicz et al. [32] reported comparable retentive force values between digitally fabricated denture bases (3D-printed and milled) when compared to conventional bases. Chebib et al. [33] reported that digitally fabricated bases (printed and milled) fabricated from conventional impressions that included the clinical steps of border-molding and posterior palatal seal compression presented significantly higher retentive forces (2-3 fold) than digitally fabricated bases (printed and milled) that were fabricated from digital impressions (intraoral scans). They added that these differences were significant in all directions of dislodgement as well as after two weeks of storage in artificial saliva.

As mentioned earlier, other studies [11,21,29] evaluated retention as well as stability (along with other factors) using clinician-based assessments. Srinivasan et al. [11] evaluated the retention and stability of maxillary and mandibular dentures as well as other items including lip support, lower lip line, and balanced occlusion. The overall clinician’s denture quality assessment revealed that both digitally fabricated dentures (milled and printed) were significantly better rated than the old conventional dentures in all evaluated parameters with insignificant differences between them. It is worth mentioning, however, that the number of mandibular dentures rated with unsatisfactory retention was lower in the milled group (n=1) as opposed to the 3D-printed group (n=4). Osnes et al. [21] also used clinician-based assessments to evaluate the retention and stability of 3D-printed and conventional dentures. 3D-printed dentures were equally as retentive as the conventional dentures in six of the 14 cases. Conventional dentures were more retentive in five cases. As for stability, the printed and conventional dentures were rated as equally stable in eight of the 14 cases. The conventional dentures were more stable in five cases, while the printed dentures were more stable in only one case. In a third study, Al-Kaff and Al Hamad [29] compared 3D-printed dentures with intraoral scanning (AMI) versus cast digitization (laboratory scanning of the definitive casts) (AMH) and conventional complete dentures (CC). A 20-year experienced prosthodontist evaluated each denture set using a 14-item assessment and a five-point Likert scale. The criteria assessed were retention, stability, and 12 other factors. Their results revealed insignificant differences among the three groups with regard to stability, fit, vertical dimension, denture base contour, lip support, phonetics, occlusion, extension, prognosis, and overall assessment. However, the CC group demonstrated significantly higher quality in tooth arrangement than the other two groups and significantly higher quality than AMI for mandibular denture retention, centric relation, and aesthetics. They also concluded that AMI-printed dentures demonstrated inferior clinical quality and retention than both AMH-printed dentures and conventional dentures, especially for the mandibular arch.

It seems that in most of these aforementioned studies, 3D-printed dentures (or denture bases) are either equivalent or fairly more retentive when compared to milled and conventional ones. The authors in these studies [11,21,26,27,32] attributed this to the effect of the CAD-CAM manufacturing process as the shrinkage that usually occurs with the conventional heat polymerization process is considerably decreased for printed bases. This, in turn, results in improved fit and better adaptation to the supporting tissues that consequently improve retention. Moreover, it seems that greater retention could be achieved when constructing 3D-printed dentures from conventionally recorded and border-molded impressions [33].

Post-insertion Maintenance

Kim et al. [23] conducted a retrospective study to investigate clinical performance and post-insertion complications of 3D-printed dentures in comparison to conventional dentures. A number of post-insertion adjustments and remakes, in addition to the type and number of repairs (including relining and rebasing), were investigated. Their results revealed insignificant differences between the two denture types in all parameters tested. The authors believed that these findings were due to the short-term one-year study period, which they considered as a limitation. They recommended that future studies with longer follow-up periods should be conducted to investigate the long-term clinical performance of printed dentures. In another relatively long-term (two and half year) study, Ohara et al. [7] compared conventional to 3D-printed dentures in terms of the number of clinical treatment visits, time required for denture fabrication, number of adjustment visits, as well as time required for denture stabilization (until participants were capable of masticating without any pain). They reported that both denture types showed comparable results for the time required for denture fabrication, number of adjustment sessions, and denture adjustments. However, the number of clinical treatment visits required for definitive denture fabrication (including the number of remakes) was significantly higher for the printed dentures. The authors explained that this unexpected finding may be attributed to the procedural errors and challenges faced in recording occlusion with digital dentures as was reported by previous studies [38-40].

Srinivasan et al. [11] investigated maintenance and adjustment requirements for digitally fabricated (milled and printed) dentures. They recorded the type of intervention, the time needed for it, and the related clinical and laboratory costs. They defined maintenance as any modification performed on the digital dentures that required laboratory procedures and adjustments referred to the needed chair-side modifications. Both were recorded whether done during a regular or an unscheduled recall visit. Their results revealed that there were no maintenance costs for the milled dentures, while three maintenance visits were recorded for the 3D-printed dentures, one during a planned visit and two required unscheduled visits, with an average time of five and 10 minutes, respectively. Moreover, the 3D-printed dentures required more clinical time for adjustments and consequently more cost than the milled dentures. These findings were not justified by the authors.

Computerized Occlusal Force Analysis

Chaturvedi et al. [22] compared occlusal force parameters in conventional dentures to digitally fabricated dentures, milled and printed, using a computerized occlusal analysis system (T-Scan III). The dentures were additionally divided into three sub-groups based on three occlusion schemes: (bilateral balanced (BBO), monoplane (MO), and lingualized (LO)). The study included a total of 45 dentures constructed for five participants (each receiving nine sets of dentures). Occlusal force analysis included the percentage of occlusal force applied on the right and left sides of the arch and the difference between them, centralization of forces, and the percentage of maximum occlusal force. Based on their results, the authors reported that both the fabrication technique and the occlusal scheme influenced the occlusal parameters investigated. They added that digital dentures were superior to conventional dentures and that BBO and LO occlusal schemes seemed to be better than MO. The authors justified the superiority of digital dentures by the fact that tooth displacement is expected during conventional processing procedures due to the problems of polymerization shrinkage, high pressure of packing, and uncontrolled expansion of plaster. The authors also reported that printed dentures with BBO and LO occlusal schemes demonstrated more centralization of forces, better force distribution, and higher percentages of maximum occlusal force. However, they stated that this could be attributed to the differences in the technique of milling and 3D printing without any further clarification.

2. Patient-based outcomes

Patient-reported outcomes have become increasingly substantial in dental research as they are capable of providing unique insights into the patient’s own experience and offer a comprehensive assessment of the provided treatment directly from the patient’s perspective. This subsequently enhances both the quality of care and clinical research. They complement the objective clinical measures, hence providing a more holistic view of treatment outcomes and patient satisfaction [41].

A number of articles mentioned earlier in this review complemented their objective clinical outcomes with subjective and patient-centered outcomes mainly through patient satisfaction questionnaires and oral health-related quality of life (OHRQoL) instruments. Most of these studies investigated patient satisfaction using a visual analog scale (VAS) [5,7,26,31,32]. The most commonly investigated items were aesthetics, speech, masticatory efficiency, hygiene (ease of cleaning), comfort, and general satisfaction. However, some studies included other items in their questionnaire such as retention [7,31,32], stability [7], pain [7,31], taste sensation [31,32], and smoothness [32]. One study [26] reported scores for general satisfaction only. Srinivasan et al. [11] used a five-point Likert scale instead of VAS to assess patient satisfaction with six of the previously mentioned items (aesthetics, speech, masticatory efficiency, stability, retention, and general satisfaction). Heikal et al. [28], on the other hand, used a specially designed questionnaire focusing on denture-related complaints and a general satisfaction rate [42]. It comprised five main domains including a functional complaint about the denture, overall masticatory ability, masticatory ability for different types of food, the effect on mental and daily life, and overall denture satisfaction. Four of the included studies assessed the OHRQoL as one of the outcomes [5,7,11,29], and they all used the oral health impact profile for edentulous patients (OHIP-EDENT) instrument.

Patient Satisfaction

Results of patient satisfaction with 3D-printed dentures varied and were inconsistent among the included studies as some reported positive results in all assessed aspects while others reported some concerns.

Cristache et al. [5] evaluated 3D-printed dentures manufactured using improved PMMA-nanosized titanium dioxide (nanoTiO_2_) and reported significant improvements in all assessed satisfaction criteria throughout the follow-up. It is noteworthy that the lowest satisfaction score was recorded for the aesthetic item after 18 months of denture use. The authors attributed this finding to the color changes that may occur intraorally to acrylic resins in general, mainly due to the slow absorption of water over time owing to the polar nature of the resin molecules. In another study, Stilwell et al. [25] investigated the extraoral appearance of conventional versus digitally fabricated dentures. Their study included four groups: dental technicians, undergraduate students, postgraduate residents, and elderly complete denture wearers. They were asked to evaluate the appearance of maxillary dentures manufactured by six different techniques (three conventional approaches: flask-pack-press, injection-molding, intrinsically colored natural gingiva finish before injection-molded; and three CAD-CAM methods: milled base with bonded prefabricated teeth, fully milled with milled teeth, printed dentures with printed teeth). Their results revealed that the lowest scores were recorded for the 3D-printed dentures for global preference, general tooth appearance, and tooth color. Dental technicians rated the 3D-printed dentures as significantly worse than the others. Denture wearers also rated the 3D-printed dentures as the worst though they were less discriminative in their judgement than the dental students and residents. 3D-printed dentures were rated the worst mainly in the tooth-related parameters (white aesthetics) compared to the other denture types. The authors recommended that future developments should concentrate on improving these parameters in order to achieve enhanced tooth color, translucency, and surface detail.

Another study conducted by Osnes et al. [21] highlighted the aesthetic challenges that faced printed dentures. Participants were asked to rate their 3D-printed and conventional dentures with regard to three main aspects: comfort, stability, and appearance. Only two out of the 14 participants (14%) were very satisfied with the appearance of the 3D-printed dentures, while eight out of 14 (57%) were very satisfied with the conventional dentures. The authors attributed the compromised satisfaction with aesthetics to the monolithic nature of 3D-printed teeth. As for comfort, all 14 participants rated their conventional dentures as comfortable to very comfortable, while only two rated their 3D-printed dentures as very comfortable and seven rated them as comfortable. For stability, nine participants rated the conventional dentures as very stable and six participants rated the 3D-printed dentures as very stable.

Nonetheless, the appearance and aesthetics of 3D-printed dentures did not seem to be a major concern in other studies. Srinivasan et al. [11] reported insignificant differences between milled and printed dentures in all satisfaction aspects including the aesthetic item. It is worth mentioning, however, that commercially available pre-fabricated artificial teeth were used. Hence, the aesthetic drawbacks of the monochromatic printed teeth were avoided. It is fair to say though that despite these insignificant differences, milled dentures demonstrated slightly higher scores than the 3D-printed dentures in all categories except in the ability to speak in which both were considerably comparable. The authors questioned, however, if these findings would differ if the study sample size was larger. Similar findings were observed by Heikel et al. [28] who also reported insignificant differences between milled, 3D-printed, and conventional dentures in all five satisfaction domains they assessed. Interestingly enough, they reported that for all satisfaction domains; the lowest satisfaction scores were recorded for the milled group followed by the conventional, while the highest satisfaction scores were for the 3D-printed group. The noticeable dissimilarity between the two aforementioned studies may be partially related to the different satisfaction questionnaires implemented in both studies.

Studies that compared 3D-printed dentures to conventional dentures revealed a concern for phonetics among other items. Ohara et al. [7] reported that conventional dentures were superior in terms of phonetics, ease of cleaning, stability, comfort, and general satisfaction. The authors attributed the inferiority of printed dentures in phonetics to the fact that they need to be fabricated with a palatal thickness of around 2.5 mm to ensure strength. On the other hand, they attributed the superiority of conventional dentures in stability and comfort to the stable occlusal contacts as well as the good peripheral seal at the denture borders. In a more recent study, Kang et al. [31] also reported that phonetics was significantly better with conventional dentures; however, there were insignificant differences between the 3D-printed dentures and the conventional dentures in all other satisfaction items. The authors also attributed this finding to the thicker palatal contours in digitally fabricated dentures. Only one of the included studies reported that patient satisfaction was higher for 3D-printed dentures compared to conventional dentures [26]. This study, however, investigated and reported general satisfaction scores only and the digital dentures were constructed for the maxillary arch only. 

Maniewicz et al. [32] compared milled and printed maxillary denture bases to conventional ones. They assessed comfort in addition to aspects different than those mentioned above, including taste, smell, fit sensation, and smoothness. They reported that, in general, participants evaluated all denture bases favorably irrespective of the fabrication method. The only exception was for the smoothness aspect where conventional bases were significantly superior to the digitally fabricated varieties. 

OHRQoL

Cristache et al. [5] reported that their 3D-printed dentures made from improved PMMA-nanoTiO2 significantly improved the OHIP-EDENT scores after 18 months of denture wearing as compared to before treatment. Srinivasan et al. [11] reported insignificant differences in OHIP-EDENT scores between the digitally fabricated dentures (milled and 3D-printed) when compared to the participants’ old conventional dentures. That was true for both the overall as well as the individual domain scores. They also reported that that the scores did not significantly change throughout the study period for both digital denture groups. Similarly, Ohara et al. [7] reported comparable oral health impact profile (OHIP) scores between 3D-printed and conventional dentures except for the social disability domain in which the 3D-printed dentures scored higher (indicating poorer OHRQoL). The authors, however, did not justify this finding.

As mentioned earlier, Al-Kaff and Al Hamad [29] compared 3D-printed dentures with intraoral scanning versus cast digitization (laboratory scanning of the definitive casts) and conventional complete dentures. Despite that, the printed dentures constructed from intraoral scanning of the edentulous arches showed slightly poorer OHIP scores than those printed following the digitized cast protocol and the conventional dentures; this was not enough to show significant differences among the three groups. It is important to mention that the participants wore each denture for three to four weeks only with a one-day washout period; hence, both the observation and washout periods were short. This was a major limitation in this study (as mentioned by the authors) as this time may not have been enough to show differences in OHIP scores among the groups.

Patient Preference and Willingness to Pay

Three studies reported patient preference among their outcomes. In the Srinivasan et al. [11] study, eight out of 15 participants preferred the milled dentures, while seven preferred the 3D-printed dentures. It was noted, however, that the milled dentures were preferred by the older participants and those who were edentulous for a longer time. Ohara et al. [7] reported that 12 participants preferred and selected the conventional dentures at the end of the study, while only three preferred the digitally fabricated dentures. Similarly, in the Osnes et al. [21] study, participants also tended to favor conventional dentures over 3D-printed ones. Eight (57%) preferred the conventional dentures, four (29 %) had no preference, and only two (14%) preferred the 3D-printed dentures.

To the best of the authors’ knowledge, only one study [11] assessed the willingness of the participants to pay for the performed treatment. Their results revealed that the participants were willing to pay more than the actual cost for both digital denture types (milled and 3D-printed); however, they were willing to pay more for the milled than for the 3D-printed dentures. The authors explained that evaluating such an outcome is important for the clinical decision-making process.

Patient Reported Complications

One study [23] assessed patient-reported complications based on symptoms and participants’ own statements. Six items were evaluated: pain and visible ulcers, simple discomfort, lack of retention, lack of stability, aesthetic problems, and occlusal problems (discomfort upon chewing or loss of retention upon occluding). Their results revealed significantly higher occurrences of pain and visible ulcers under the conventional dentures (46.67% and 55.33% in maxilla and mandible, respectively) compared to the printed dentures (36.15% and 34.88% in maxilla and mandible, respectively). There were insignificant differences between the maxillary dentures of both groups in all other items. As for the mandibular dentures, the conventional group showed higher discomfort (30.67%) versus the printed group (17.44%). On the contrary, aesthetic complications were higher in the printed group (11.63%) when compared to the conventional group (4.67%). The authors justified the superiority of printed dentures with regard to pain, visible ulcers, and discomfort by the improved internal fit achieved by such dentures. They also explained the superiority of conventional dentures in aesthetics by the monochromatically colored nature of the teeth used with printed dentures as compared to the multi-layered colored teeth used with conventional dentures.

Limitations of this review included the restriction to studies published in English. Only the most common databases were utilized. Some of the studies were with a limited sample size and were not double-blinded. The risk of bias and homogeneity of the included studies were not assessed. As a narrative review, concrete guidelines could not be concluded; rather, an overview of the available literature has been presented. Considering the differences in the methodologies and the small sample size of some articles, more randomized clinical studies with larger sample sizes and standardized methods are recommended.

## Conclusions

3D printing of complete dentures seems to offer many advantages from both a clinical and patient-based perspective. Clinically, it was found that retention and comfort with 3D-printed dentures are comparable or even superior to conventional dentures. However, retention of 3D-printed dentures constructed from a protocol adopting intraoral scanning (digital impressions) demonstrated inferior retention when compared to those constructed from conventional impressions and digitized casts. Results were somewhat variable with regard to masticatory efficiency and biting force. As for post-insertion maintenance, printed dentures showed comparable results to conventional dentures in the short term. However, more studies with longer follow-up periods are recommended to investigate their long-term clinical performance.

Subjectively, ORHQoL and patient satisfaction with 3D-printed dentures were slightly inconsistent among the included studies. Most of the studies reported positive results in the different domains and assessed aspects while others reported some concerns (especially in terms of aesthetics and phonetics). The technique seems promising with numerous benefits, however, further clinical research with larger sample sizes and longer follow-up periods is still needed to confirm these conclusions and address the potential concerns.
